# Structural analyses of the GI.4 norovirus by cryo-electron microscopy and X-ray crystallography revealing binding sites for human monoclonal antibodies

**DOI:** 10.1128/jvi.00197-24

**Published:** 2024-04-09

**Authors:** Tomomi Kimura-Someya, Kazushige Katsura, Miyuki Kato-Murayama, Toshiaki Hosaka, Tomomi Uchikubo-Kamo, Kentaro Ihara, Kazuharu Hanada, Shin Sato, Kazutaka Murayama, Michiyo Kataoka, Mikako Shirouzu, Yuichi Someya

**Affiliations:** 1 RIKEN Center for Biosystems Dynamics Research, Yokohama, Kanagawa, Japan; 2 Graduate School of Biomedical Engineering, Tohoku University, Sendai, Miyagi, Japan; 3 Department of Pathology, National Institute of Infectious Diseases, Tokyo, Japan; 4 Department of Virology II, National Institute of Infectious Diseases, Tokyo, Japan; University of Kentucky College of Medicine, Lexington, Kentucky, USA

**Keywords:** norovirus, cryo-electron microscopy, virus-like particle, human monoclonal antibody, scFv, Fv-clasp, X-ray crystallography, VP1, P domain

## Abstract

Noroviruses are major causative agents of acute nonbacterial gastroenteritis in humans. There are neither antiviral therapeutic agents nor vaccines for noroviruses at this time. To evaluate the potential usefulness of two previously isolated human monoclonal antibody fragments, CV-1A1 and CV-2F5, we first conducted a single-particle analysis to determine the cryo-electron microscopy structure of virus-like particles (VLPs) from the genogroup I genotype 4 (GI.4) Chiba strain uniformly coated with CV-1A1 fragments. The results revealed that the GI.4-specific CV-1A1 antibody bound to the P2 subdomain, in which amino acids are less conserved and variable. Interestingly, a part of the CV-1A1 intrudes into the histo-blood group antigen-binding site, suggesting that this antibody might exert neutralizing activity. Next, we determined the crystal structure of the protruding (P) domain of the capsid protein in the complex form with the CV-2F5 antibody fragment. Consistent with the cross-reactivity, the CV-2F5 bound to the P1 subdomain, which is rich in amino acids conserved among the GI strains, and moreover induced a disruption of Chiba VLPs. These results suggest that the broadly reactive CV-2F5 antibody can be used as both a universal detection reagent and an antiviral drug for GI noroviruses.

**IMPORTANCE:**

We conducted the structural analyses of the VP1 protein from the GI.4 Chiba norovirus to identify the binding sites of the previously isolated human monoclonal antibodies CV-1A1 and CV-2F5. The cryo-electron microscopy of the Chiba virus-like particles (VLPs) complexed with the Fv-clasp forms of GI.4-specific CV-1A1 revealed that this antibody binds to the highly variable P2 subdomain, suggesting that this antibody may have neutralizing ability against the GI.4 strains. X-ray crystallography revealed that the CV-2F5 antibody bound to the P1 subdomain, which is rich in conserved amino acids. This result is consistent with the ability of the CV-2F5 antibody to react with a wide variety of GI norovirus strains. It is also found that the CV-2F5 antibody caused a disruption of VLPs. Our findings, together with previous reports on the structures of VP1 proteins and VLPs, are expected to open a path for the structure-based development of antivirals and vaccines against norovirus disease.

## INTRODUCTION

Noroviruses, which are classified in the family *Caliciviridae*, are the leading causative agents of nonbacterial acute gastroenteritis in humans (1). Among 10 genogroups of noroviruses (GI to GX) identified, GI and GII noroviruses are the major pathogens for humans, and are further classified into 9 and 27 genotypes, respectively (2, 3). Almost 90% of human noroviruses isolated from patients belong to GII, and the remaining 10% consist of GI, GIV, and GIX noroviruses (4–7). Not all genotypes are equally isolated worldwide, and epidemic genotypes vary season-by-season, with the exception that GII.4 noroviruses have always been dominant, sometimes with minor modifications that are recognized as subtypes or variants. Like novel GII genotypes (3), emerging genotypes in both GI and GII might evoke outbreaks in humans since considerable numbers of humans, especially infants and young children, are thought to be immunologically naïve against them.

The norovirus capsid is composed of 180 molecules of the VP1 major capsid protein which is encoded by open reading frame 2 (ORF2) in the positive-sense, single-stranded RNA genome. Normally, 90 dimers of VP1 proteins self-assemble to form a T = 3 icosahedral particle of 38 nm in diameter (8). ORF3 encodes a minor structural protein, VP2, which is rich in basic amino acids, and it was found that the VP2 protein was associated with the shell domain of the VP1 protein within the virus particle (9). It was recently shown that the VP2 protein of feline calicivirus formed a portal-like assembly after its receptor binding, which possibly functioned as a genome translocating channel (10). When the norovirus ORF2 gene, even without the ORF3 gene, is expressed in insect cells via recombinant baculoviruses, virus-like particles (VLPs) without the genome are formed and excreted in culture media (11). Although 38 nm VLPs are a major product, sometimes 23 nm VLPs happen to be produced (12, 13). Smaller particles are known to have a T = 1 icosahedral symmetry (14).

The crystal structure of 38 nm VLPs from the GI.1 Norwalk strain was solved for the first time in 1999 (8), showing that the VP1 protein consists of two domains: an S domain that forms a contiguous spherical shell, and a P domain that protrudes from the shell. The P domain is further divided into two subdomains, P1 and P2. Compared to the P1 subdomain, amino acids in the P2 subdomain are less conserved, which is attributed to the presence of a wide variety of genotypes and thus the differences in antigenicity. The crystal structure also revealed that the P2 subdomain resided on the outmost surface of the virus capsid (8).

It is known that 23 nm particles are excreted in human feces (15) as well as in an *in vitro* culture using human enteroids (16). In 2019, Jung et al. revealed the cryo-electron microscopy (EM) structures of the GI.1 Norwalk and GI.7 TCH060 VLPs with a T = 3 icosahedral symmetry, T = 1 and T = 3 VLPs from the GII.2 Snow Mountain strain, and, most strikingly, a T = 4 VLP from the GII.4 Minerva strain (17). Their findings indicated that norovirus VP1 proteins are capable of forming VLPs with various types of icosahedral symmetry; however, it remains unknown whether virions with a T = 4 architecture exist in nature.

The GI.4 genotype has been one of the major genotypes in genogroup I for many years in Japan (7), and the Chiba strain, a prototype for GI.4, has thus been extensively studied (13, 14, 18–20). It is noteworthy that the GI.4 Chiba strain has recently been investigated as a bivalent vaccine candidate together with the GII.4 VLPs (21). Therefore, insights into the antigenic structure could inform vaccine development. Higo-Moriguchi and colleagues (22) isolated several monoclonal antibodies (mAbs) in the form of single-chain variable fragments (scFvs) from a human peripheral blood library by using the GI.4 Chiba VLPs as bait. One of the scFvs, the CV-1A1, is highly specific to the GI.4 VLPs, and it strongly inhibits the binding of the Chiba VLPs to histo-blood group antigens (HBGAs). Experiments conducted using a stem cell-derived enteroids system have shown that the potency of microneutralization correlates with HBGA blockade of polyclonal sera and mAbs (23–26). It is therefore suggested that the CV-1A1 antibody might have neutralizing activity against GI.4 strains because HBGAs are implicated as a potential cellular attachment factor at the initial step of norovirus infection. Another mAb, the CV-2F5 scFv, has a broad spectrum of reactivity; it reacted not only with the GI.4 Chiba VLP, but also with the GI.1 Aichi124-89, GI.2 Funabashi258, GI.3 Kashiwa645, and GI.6 WUG1 VLPs [note that the WUG1 strain used was formerly classified in GI/8, which was incorrectly written as GI.8 in reference (22)]. Epitopes for these antibodies remain unknown.

In the present study, we conducted a cryo-EM analysis of VLPs from the GI.4 norovirus and an X-ray crystallographic analysis of the *P* domain from the VP1 protein in order to determine the structural basis for the recognition of the respective epitopes by human mAbs. Our analyses revealed that the epitope of CV-2F5 is localized in the P1 subdomain, which is the region conserved in the GI noroviruses, and the epitope of CV-1A1 is in the P2 subdomain, which is variable among the norovirus genotypes.

## RESULTS

### Cryo-EM analysis of the Chiba VLPs with antibody fragments

**Fig 1:**
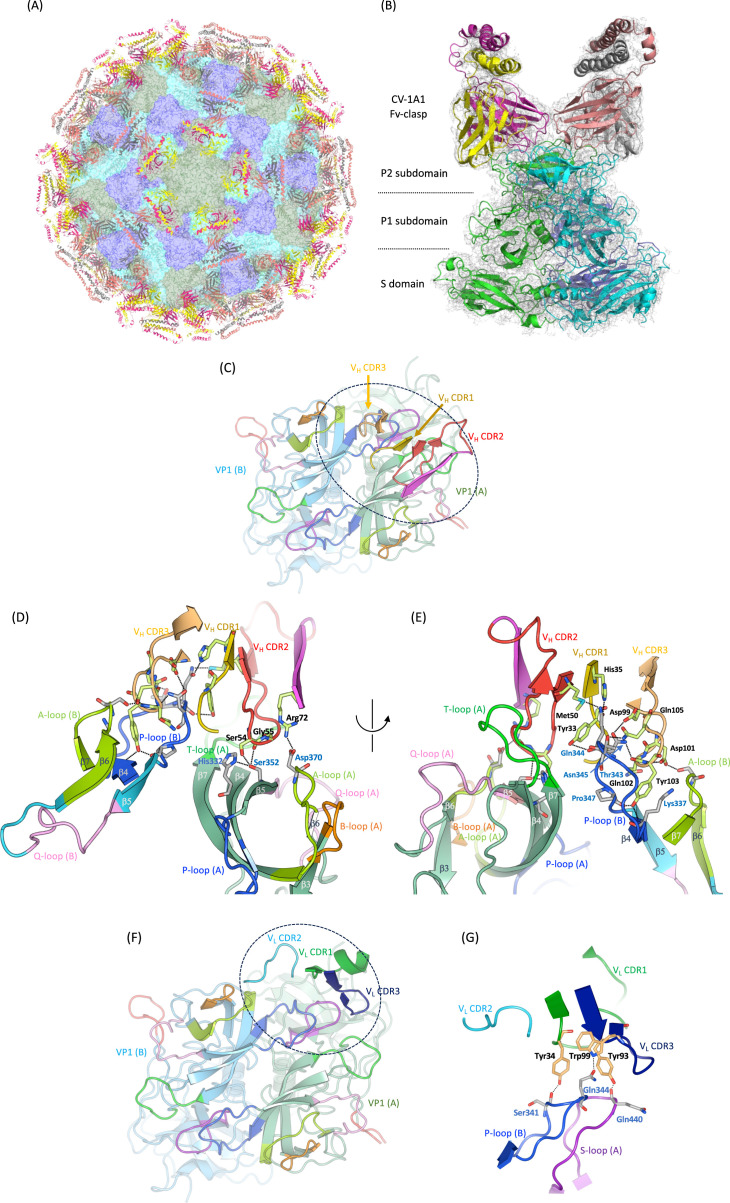
Cryo-EM structure of the GI.4 Chiba VLP complexed with the CV-1A1 Fv-clasp. (**A**) The overall structure of the complex. The surfaces of the A, B, and C subunits of the VP1 protein are colored green, cyan, and blue, respectively. The CV-1A1 Fv-clasp antibodies are shown in a ribbon model. Antibodies bound mainly to the A subunits of the VP1 proteins are marked in magenta for the V_H_ domain and yellow for the V_L_ domain, and those bound mainly to the B subunit are marked in pale red for the V_H_ domain and gray for the V_L_ domain. (**B**) The structure of the CV-1A1 Fv-clasp-bound VP1 proteins. Each of the proteins is colored in the same way as in panel (**A**). (**C–E**) The binding interface between the P2 subdomain and the V_H_ domain of the CV-1A1 Fv-clasp. On panel (**C**), the VP1 A/B dimers are viewed from above, and only the V_H_ CDRs of antibodies mainly bound to the A subunit are shown. The region encircled with a dotted line is magnified on panels (**D** and **E**). The structures on panels (**D** and **E**) were viewed from different angles. (**F and G**) The binding interface between the P2 subdomain and the V_L_ domain of the CV-1A1 Fv-clasp. On panel (**F**), the VP1 A/B dimers are viewed from above, and only the V_L_ CDRs of antibodies mainly bound to the A subunit are shown. The region encircled with a dotted line is magnified on panel (**G**). On panels (**D**), (**E**), and (**G**), the amino acid residues mentioned in the main text are shown in a stick model, and indicated using three-letter codes with the number of each position (blue letters for the VP1 protein and black letters for the CV-1A1 Fv-clasp). Sticks of residues forming the VP1 protein, V_H_ CDRs, and V_L_ CDRs are colored pale gray, yellow green, and pale orange, respectively.

To evaluate the accessibility of two antibody fragments, CV-1A1 and CV-2F5, which were isolated by Higo-Moriguchi et al. (22), to VLPs as surrogates for infectious viruses, we attempted to analyze the structure of the VLPs by cryo-EM. The CV-1A1 scFv is highly specific to the Chiba VP1 protein. An Fv-clasp fragment has elevated thermal stability as well as a tendency to form a tightly bound Fv dimer (27). We therefore prepared the Fv-clasp version of CV-1A1. The CV-1A1 Fv-clasp fragments were mixed with the Chiba VLPs, and the cryo-EM structure of the VLP-Fv-clasp complex was solved as shown in Fig. 1. The CV-1A1 Fv-clasp antibodies bind to the P2 subdomain composing the outermost layer of the virus particles, and one Fv-clasp molecule exists on the A/B dimer of VP1 proteins without interference from the neighboring C/C dimers. As shown in Fig. 1, the CV-1A1 antibody uses mainly complementarity-determining regions (CDRs) of its V_H_ domain to recognize the GI.4 VP1 protein, namely at the binding interface, three CDRs from one molecule of the Fv-clasp recognize the T-, Q-, and A-loops from one protomer of the VP1 protein and the A- and P-loops from another protomer.

**Fig 2:**
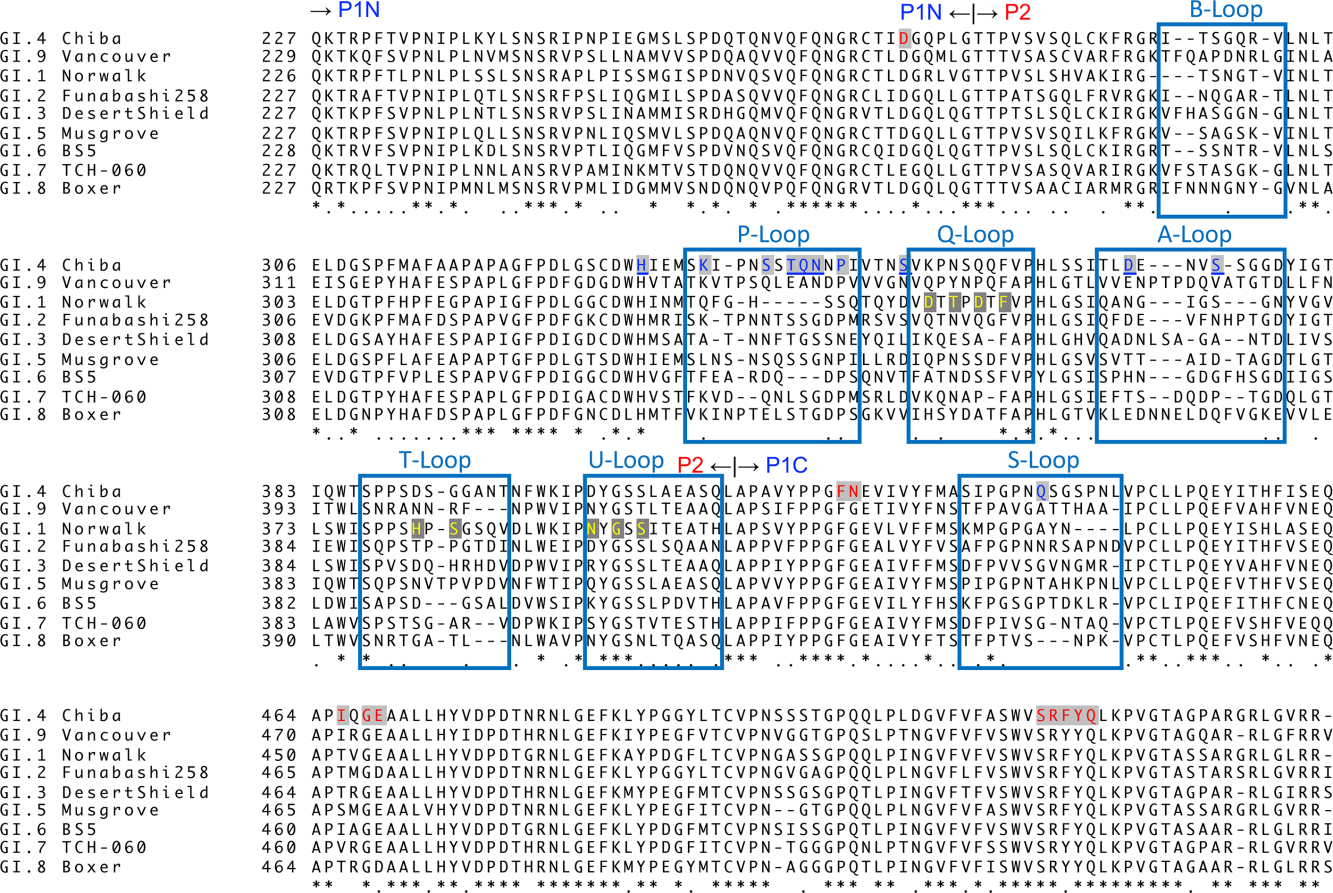
Amino acid alignment of VP1 proteins from the GI noroviruses. Amino acid sequences of the P1 and P2 subdomains were aligned with the help of GENETYX-MAC software (Genetyx Corp., Tokyo). “P1N” and “P1C” indicate the N-terminal and C-terminal parts of the P1 subdomain, respectively. Asterisks (*) indicate positions at which amino acid residues are conserved, and periods (.) indicate positions occupied by similar amino acids. The roles of each residue of the GI.4 Chiba VP1 are represented with colored letters on a gray background as follows: blue letters for the binding of the CV-1A1 Fv-clasp; red letters for the binding of the CV-2F5 scFv. The side chains of underlined residues are responsible for the CV-1A1 binding. For comparison, amino acid residues of the GI.1 Norwalk VP1 involved in binding the IgA 5I2 Fab (19) are indicated by yellow letters on a gray background. Loop regions are boxed with their respective names.

Interestingly, several residues on the P-loop and His322 on the β4-sheet prior to the P-loop are thought to be involved in the binding of HBGA, based on the structural analysis of the related GI.9 VP1 protein (28). In contrast, the contribution of the V_L_ domain is rather minor, with CDR1 and CDR3 recognizing the P-loop from one protomer and the S-loop from another protomer. Amino acid residues responsible for the binding of the CV-1A1 antibody are shown in Fig. 2.

We considered that it would be interesting to know whether and how the CV-1A1 antibody affects the HBGA binding to the VP1 protein. Since there is currently no available structural information for any complex formed by the Chiba VP1 protein or its P domain protein and any HBGA, we used the structure of the Lewis b-bound GI.9 Vancouver P domain protein (28) to assess the structure of a Chiba VP1–HBGA complex. When we superimposed it with the structure of the CV-1A1-bound Chiba VP1 protein, we observed that the CDR2 from the V_H_ domain (hereafter designated V_H_ CDR2) intruded into the Lewis b-binding site (Fig. S1). This observation is well consistent with the previous finding that the CV-1A1 scFv strongly inhibited the binding of the Chiba VLPs to HBGAs (22). Together, these findings suggest that the CV-1A1 antibody will effectively inhibit the binding of GI.4 noroviruses to HBGAs and/or a possible receptor molecule.

### The X-ray structure of the complex of the P dimers with the GI-broadly reactive mAb fragment CV-2F5

**Fig 3:**
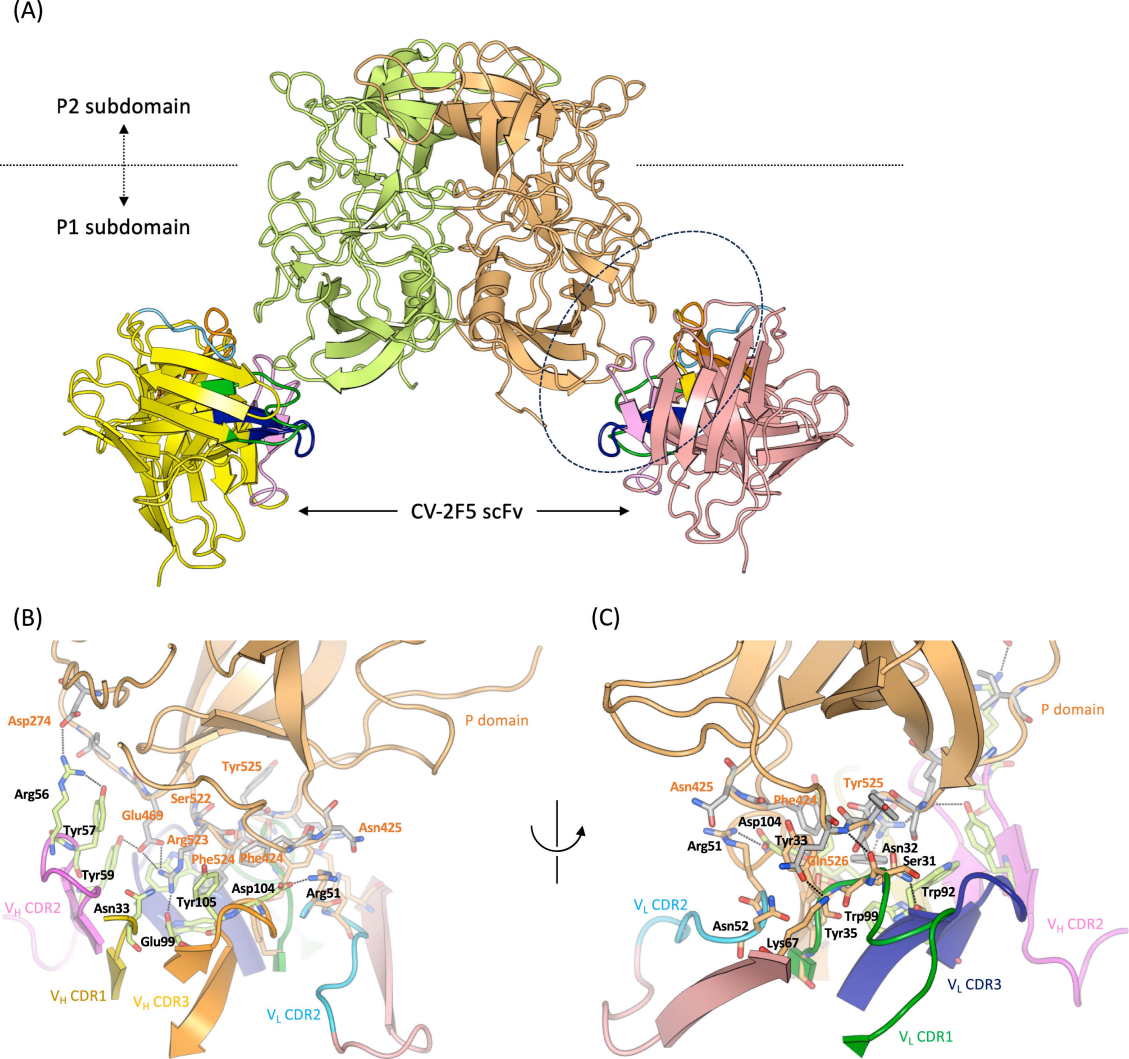
Crystal structures of P dimers of the GI.4 Chiba VP1 protein complexed with the CV-2F5 scFv. (**A**) The overall structure of the complex. The CV-2F5 scFv binds to the P1 subdomain of the P dimer. The region encircled with a dotted line was magnified on panels (**B) and (C**). (**B and C**) The binding interface between the P1 subdomain and the CV-2F5 scFv. The structures on panels (**B**) and (**C**) were viewed from different angles. Amino acid residues mentioned in the main text are shown in a stick model, and indicated using three-letter codes with the number of each position (orange letters for the VP1 protein and black letters for CV-2F5 scFv). Sticks of residues forming the VP1 protein, V_H_ CDRs, and V_L_ CDRs are colored pale gray, yellow green, and pale orange, respectively.

In contrast to the CV-1A1 antibody, the CV-2F5 antibody is GI-broadly reactive (22), and it would thus be intriguing to investigate the binding property of the CV-2F5 on the norovirus. We attempted to isolate the CV-2F5-bound VLPs, but the attempt was not successful (the reason is described in the next section). We then changed the strategy to crystallography. The Chiba P dimers were co-crystallized with the purified CV-2F5 scFv antibodies. The X-ray structure of the complex was determined at 2.7 Å resolution (Fig. 3), revealing that CV-2F5 scFv bound tightly to the P1 subdomain of the Chiba P dimers. It is likely that the epitope of the CV-2F5 antibody consists mainly of three parts. The first part is the stretch from Pro422 to Glu426 preceding beta 8 and the S-loop, which are recognized by Asp104 in the V_H_ CDR3, Tyr33 in the V_L_ CDR1, and Arg51 in the V_L_ CDR2. The second part is three residues (Ile466, Gly468, and Glu469) preceding beta 10, which are recognized by the V_H_ CDR2. The third part is the sequence from Val521 to Leu527 in the last part of the P1C subdomain, which is recognized by the V_H_ CDR3, V_L_ CDR1, and V_L_ CDR3. It seems likely that Lys67 on the beta-sheet following V_L_ CDR2 participates in hydrogen bonding with Gln526 in the P1C subdomain. In addition, Asp274 in the P1N subdomain and Pro491 and Val498 in the P1C subdomain contribute to the antibody binding. The sequence alignment revealed that these amino acid residues (except Ile466) are highly conserved in the GI noroviruses (Fig. 2). The antibody binding hardly affected the position of the main chain of the P domain compared to the structure of the Chiba P dimer in the apo form (data not shown).

When the Chiba P domain/CV-2F5 scFv complex structure was overlaid onto the cryo-EM structure of the Chiba VLP, we observed that the CV-2F5 scFv clashed with the S domain of the VP1 protein (Fig. S2). This result raises the possibility that CV-2F5 scFv is not accessible to the epitope in the inner side of an intact VLP, at least in this conformational state. This would explain why we did not succeed in preparing the CV-2F5-bound VLPs.

### Biochemical evaluations of antibody fragments

**Fig 4:**
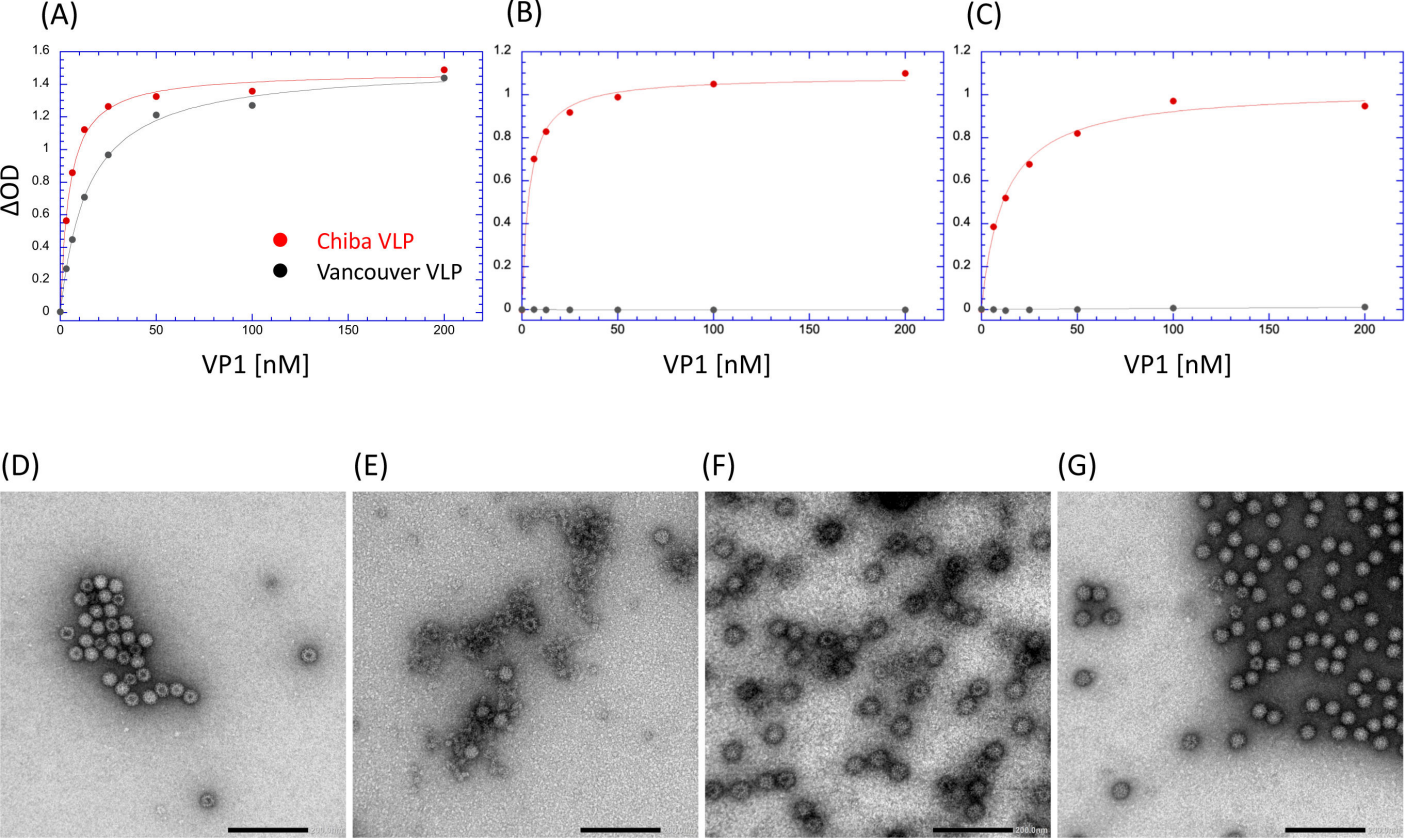
Binding of antibody fragments to VLPs and their effects on morphology of VLPs. (A – C) Antibody fragments were characterized by sandwich ELISA detecting VLPs (or VP1 proteins). VLPs were added onto microplates coated with CV-2F5 scFv (**A**), CV-1A1 Fv-clasp (**B**), or CV-1A1 scFv (**C**) as described in Materials and Methods, and the bound Chiba VLPs or VP1 proteins were detected with rabbit antisera raised using Chiba VLPs as an immunogen. The horizontal axis represents the concentration of the VP1 proteins although VLPs were used for assays. (D – G) Effects of antibody fragments on morphology of Chiba VLPs were visualized by transmission electron microscopy. As described in Materials and Methods, Chiba VLPs were incubated in the absence of antibody fragments (**D**) or in the presence of CV-2F5 scFv (**E**), CV-1A1 Fv-clasp (**F**), or CV-1A1 scFv (**G**).

To confirm the reactivity of antibody fragments prepared in this study, we conducted a sandwich enzyme-linked immunosorbent assay (ELISA) using the fragments as capture antibodies. The CV-2F5 scFv successfully trapped both Chiba VP1 proteins and Vancouver VP1 proteins (Fig. 4A), while the CV-1A1 Fv-clasp (and also the CV-1A1 scFv) reacted with only the Chiba VP1 proteins (Fig. 4B and C). These results confirmed that the CV-1A1 antibody was specific to the GI.4 VP1 proteins. Moreover, we found that the CV-2F5 scFv was able to react with the GI.9 VP1 proteins that were not tested in the previous report (22). It was also found that the CV-2F5 scFv has higher affinity for the Chiba VP1 than the Vancouver VP1 (Table 1). This might reflect that the CV-2F5 scFv was isolated by using the Chiba VP1 proteins as bait (22). As for the CV-1A1 antibody fragment, the Fv-clasp form has higher affinity than the scFv form. This observation may be consistent with the general feature that an Fv-clasp has greater stability than an scFv (27).

**TABLE 1:** Parameters for the VP1-antibody interaction

Antibody fragments	GI.4 Chiba VP1		GI.9 Vancouver VP1	
	*K_d_* (nM)	∆OD_max_	*K_d_* (nM)	∆OD_max_
CV-2F5 scFv	4.58 ± 0.39	1.48 ± 0.03	14.39 ± 0.93	1.52 ± 0.03
CV-1A1 Fv-clasp	3.77 ± 0.37	1.09 ± 0.02	–	–
CV-1A1 scFv	11.81 ± 1.25	1.03 ± 0.03	–	–

^*a*^ The data of the sandwich ELISA (Fig. 4) were fit to the Michaelis-Menten equation with the use of the KaleidaGraph software package. The Michaelis constant (*K_m_*) is considered the binding affinity (or avidity) of antibody fragments for the respective VP1 protein, and it is therefore represented as the dissociation constant (*K_d_*) in these data sets. *∆*OD_max_ corresponds to the *V_max_* value in the original equation. The parameters in this table are expressed with the respective standard errors.

Next, we assessed the effects of antibody fragments on the Chiba VLPs and Vancouver VLPs. When each VLP was mixed with a fivefold molar excess of the CV-2F5 scFv for the VP1 molecules, a portion of it was disrupted (Fig. 4E; Fig. S3B). Considering this observation, the results shown in Fig. 4A indicate that CV-2F5 scFvs coated on microplates might trap the VP1 proteins and partially dissociated VLPs with the epitope region exposed. On the other hand, the CV-1A1 of both the Fv-clasp and scFv forms did not cause a disruption of the Chiba VLPs despite binding to the Chiba VLPs (Fig. 4F and G).

**Fig 5:**
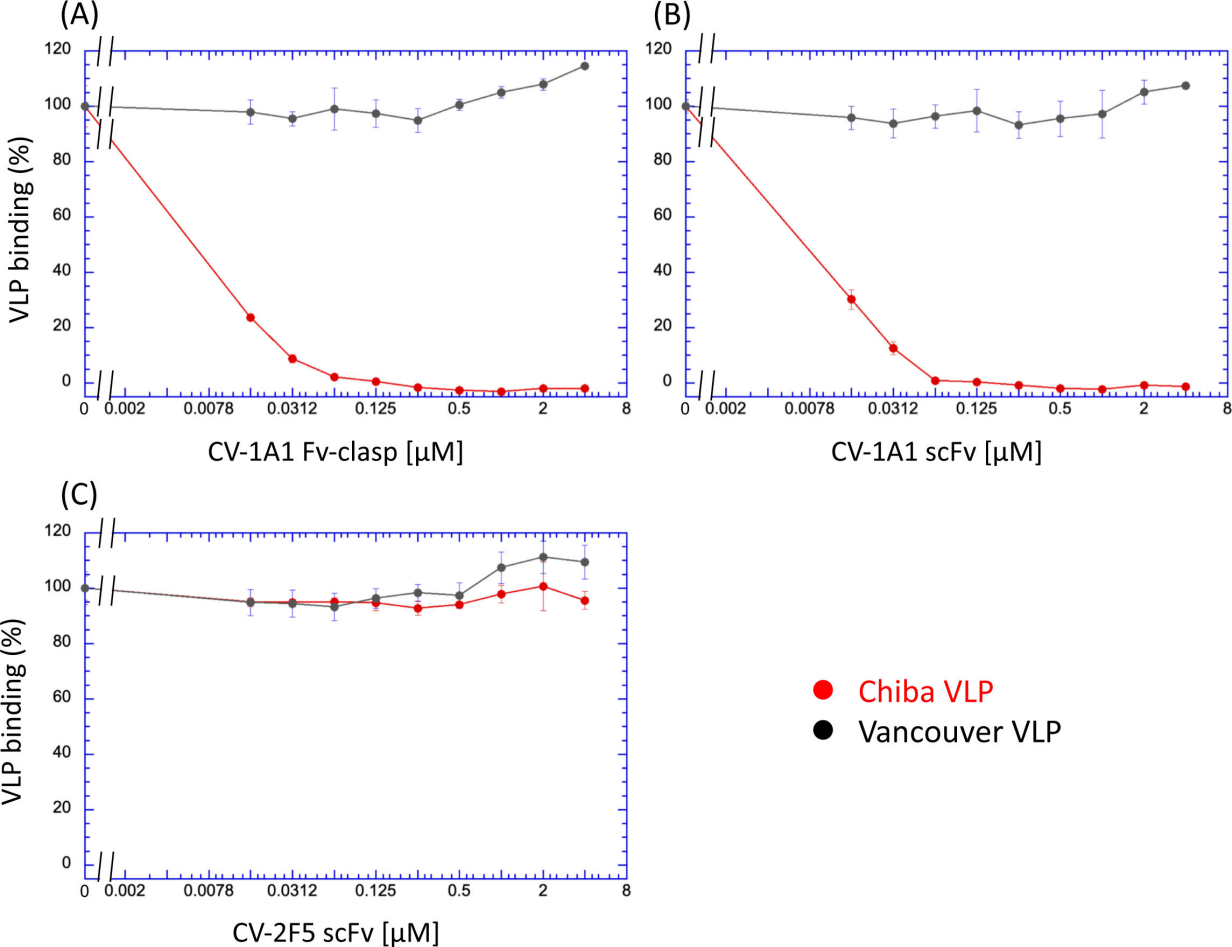
Effects of antibody fragments on VLP binding to Lewis b saccharide. Microplates were coated with the bovine serum albumin (BSA) conjugates of LNFDH I (lacto-N-difucohexaose I, Lewis b hexasaccharide) which contains Lewis b tetrasaccharide as described under the Materials and Methods, and VLPs (final concentration of 0.1 µM) and the indicated concentrations of antibody fragments [CV-1A1 Fv-clasp (**A**), CV-1A1 scFv (**B**), or CV-2F5 scFv (**C**)] were added, followed by detection of bound VP1 proteins using the anti-Chiba VLP rabbit antisera.

To further characterize these antibody fragments, we investigated whether they inhibited the binding of VLPs to HBGAs. Here, we adopted an ELISA-based binding assay in which the Lewis b antigens are coated as ligands for VLPs, as described previously (20). In the presence of the CV-1A1 Fv-clasp or the CV-1A1 scFv, the binding of the Chiba VLPs to Lewis b antigens was inhibited dependent upon the concentration of antibody fragments added, but the binding of the Vancouver VLPs was never affected (Fig. 5), confirming the specificity of the CV-1A1 antibody. These observations are well consistent with the result that the binding site of the CV-1A1 antibody overlaps the HBGA binding site in the cryo-EM structure. The 50% inhibitory concentration for the interaction between the Chiba VLPs and the Lewis b antigens was estimated to be less than 10 nM for both the CV-1A1 Fv-clasp and the CV-1A1 scFv; these values appeared to be comparable to the *K_d_* values calculated from the VLP-antibody binding assays (Table 1). In contrast, the CV-2F5 scFv never inhibited the binding of the Chiba VLPs or that of the Vancouver VLPs (Fig. 5). Here, we favor the term “VP1 proteins” instead of “VLPs,” because we observed that the CV-2F5 scFv destroyed the VLPs (Fig. 4E). Since clearly the CV-2F5 scFv reacted with both the Chiba VP1 and the Vancouver VP1 (Fig. 4A), these results strongly indicate that the binding site of this antibody fragment is different from the HBGA binding site, and that the antibody binding does not affect the binding of VLPs to HBGAs. We further found that the anti-Lewis b monoclonal antibody, but not the anti-Lewis a monoclonal antibody, inhibited the interaction between VLPs and the Chiba VP1–HBGA complex Lewis b antigens (Fig. S4). This observation reconfirms that VLPs interacted with Lewis b antigens. Moreover, this interaction was inhibited by free Lewis b tetrasaccharide at a level of mM, indicating that the affinity of VLPs for this saccharide is quite low.

## DISCUSSION

We determined the cryo-EM structures of VLPs from the GI.4 Chiba strain of norovirus in conjunction with the crystal structures of its P domain proteins, and we assessed how human monoclonal antibody fragments isolated in an earlier study (22) bound to the VP1 proteins. Since the Chiba VLPs suspended in water were somewhat fragile and some of the particles were cracked, as described previously (13), we first attempted to obtain stable VLPs with elevated integrity. Because Ausar et al. (29) previously showed that an acidic buffer may stabilize VLPs, we attempted to use 20 mM sodium citrate buffer (pH 4.2) for preparation of the Chiba VLPs. This enabled us to produce good-quality VLPs. Following the CsCl density gradient, passing through the sucrose density gradient further improved the quality of the VLPs, which were finally suspended in 20 mM sodium phosphate buffer (pH 6.8). In this way, we succeeded in preparing Chiba VLPs without obvious damage to their morphology (Fig. 1D).

### The structural basis for the binding of a GI.4-specific human mAb

The CV-1A1 antibody fragment is specific to the GI.4 strains (22). To visualize how the CV-1A1 antibody binds to a norovirus particle, we solved the structure of the CV-1A1 Fv-clasp-bound Chiba VLP. Fv-clasp fragments bound to the top of the P domain and uniformly covered the surface of the VLP without any interference between the neighboring Fv-clasp molecules (Fig. 1). A part of the binding site of the CV-1A1 antibody overlaps with that of the GI.1-specific IgA 5I2 reported by Shanker et al. (30), but the binding mode is different, that is, the orientation to the respective P2 subdomains described by Shanker et al. differs by ~180° (Fig. S5).

Notably, the CDR2 of the V_H_ domain of the CV-1A1 antibody overlaps the Lewis b tetrasaccharide when the structure of the antibody-bound Chiba VP1 protein is superimposed with that of the Lewis b-bound Vancouver P domain protein (28). We thus estimate that the CV-1A1 antibody directly interferes with the binding of noroviruses to HBGA. Several reports indicate that antibodies targeting the region near the HBGA binding pocket have neutralizing activities (26, 31–33). Based on these evidences, our present and the previous findings that CV-1A1 strongly inhibited the HBGA binding to the GI.4 Chiba VLPs [Fig. 4 and also reference (22)] support the idea that the CV-1A1 antibody is a neutralizing antibody.

In their review, Smith and Smith stated that caliciviruses including noroviruses could adopt at least two possible states, “compressed” and “expanded,” which were attributed to the flexibility of the P domains (34). Independently, Song et al. demonstrated that the P domains of murine noroviruses could adopt two (meta)stable states, the “rising” and “resting” conformations, and they suggested that these noroviruses undergo dynamic rotation between these two states in response to the solvent condition (35). These two states correspond to the “compressed” and “expanded” states, respectively. Song et al. contended that the binding of a murine norovirus to its receptor CD300lf is allowed when the virus is in the “resting” state (35). In the present study, the conformation of the Chiba VLP with the CV-1A1 antibodies which we solved was in the “resting” state (Fig. 1). This result indicates that the CV-1A1 antibody is capable of binding to VLPs when they are in the “resting” state.

Collectively, the results described in these previous reports (34–36) suggest that norovirus particles undergo dynamic conformational change depending on alterations in pH and the presence of various salts in local environments. Such flexibility would be required for virus particles, because viruses must experience more dynamic structural change for a genome release event following receptor engagement, as observed for feline calicivirus (10).

### The structural basis for the binding of a broadly reactive human mAb

The CV-2F5 scFv was isolated as a broadly reactive antibody, but its epitope has not been determined (22). Our present X-ray structural analysis of the co-crystals of this antibody fragment and the P dimer from the Chiba strain revealed that the CV-2F5 scFv bound to the P1 subdomain of the P dimer with a stoichiometry of 1:1 (two molecules of scFv for a P dimer) (Fig. 3). This is reminiscent of the broadly reactive monoclonal antibody against the GII.10 norovirus (37). Higo-Moriguchi et al. (22) used Chiba VLP solution to coat the surface of microplates, resulting in the isolation of the CV-2F5 scFv from the phage library. The fact that the epitope for this antibody is embedded and inaccessible from the outside of the particle evokes the idea that a portion of the VLPs were disrupted when adsorbed onto the microplate. The epitope of the CV-2F5 scFv was comprised of amino acids that are highly conserved among the GI noroviruses (Fig. 2), raising the possibility that this antibody is useful as a detection reagent. According to Shanker et al. (30) and others (38–40), most of the HBGA-blocking monoclonal antibodies seemed likely to be genotype-specific without cross-reactivity. Considering that (i) CV-2F5 scFv binds to the P1 subdomain which is far from the HBGA binding site formed mainly by amino acids from the P2 subdomain, and (ii) this scFv does not cause a significant conformational change in the P domain, it would be impossible that this scFv directly blocks the HBGA binding and neutralizes the GI noroviruses. However, Alvarado et al. (41) isolated broadly cross-reactive monoclonal antibodies that were able to inhibit the binding of virus particles to HBGA. In particular, they found that the NORO-320 Fab is a unique antibody that is capable of neutralizing GII.4 norovirus although it does not block the HBGA binding (41).

Superimposing the structure of the Chiba P domain with the CV-2F5 scFv on that of the VP1 with the CV-1A1 Fv-clasp, we can see that the CV-2F5 scFv overlaps with the S domain of the VP1 protein. At a glance, this would suggest that the CV-2F5 antibody might not be able to access the epitope located on the inside of virus particles in the “resting” conformation (Fig. 1). Hu et al. recently reported that the NORO-320 monoclonal antibody bound to the P domain of the VLPs from the GII strains and could exert neutralizing activity when the virus particles were in the rising conformation where their epitope was fully exposed (36).

To evaluate whether this is the case for CV-2F5 scFv, we modeled the Chiba VLP consisting of the VP1 proteins in the “rising” conformation, but nevertheless, the epitope was not exposed to permit the binding of CV-2F5 scFv (data not shown). Even so, since it has been established that this antibody fragment induces the disruption of VLPs, it may be that CV-2F5 scFv squeezes into and eventually induces the disassembly of norovirus particles, resulting in the inactivation. This would be analogous to the nanobodies isolated by Koromyslova and Hansman (42), which bound to the P1 subdomain and facilitated the disassembly of GII VLPs. These antibodies would therefore be potential antivirals.

Our data suggest that the GI.4-specific CV-1A1 human monoclonal antibody is a neutralizing antibody, and this is supported by our observation that the CV-1A1 binding site overlaps with the HBGA binding site as well as the feature that CV-1A1 scFv inhibited HBGA binding of the Chiba VLPs (Fig. 4). Although this possibility, together with the possibility described above that the CV-2F5 induces the disassembly of GI VLPs (Fig. 4), should be evaluated using organoids derived from human intestinal stem cells and infectious GI noroviruses, we have not yet obtained the necessary infectious materials. A preliminary experiment using an original stool specimen including the Chiba noroviruses (18) failed to propagate progeny viruses in human organoids.

Taken together, our present structural studies and those from other groups (30–33, 36, 37, 41, 42) have provided insight into the interaction between the VP1 proteins and antibodies, revealing that virus infection induces the production of several types of inactivating (neutralizing) antibodies against noroviruses. Based on our present analysis, genotype-specific antibodies such as the CV-1A1 antibody, which targets the P2 subdomain of the VP1 protein, prevent a virus particle from binding to HBGA and/or a possible proteinaceous receptor. In contrast, cross-reactive antibodies such as the CV-2F5 antibody bind to the P1 subdomain and do not always affect the HBGA binding, but their binding ultimately disassemble the VLPs. The latter case may be an ideal scenario in which the antibodies induced by vaccines respond to invading viruses, because not all norovirus genotypes will be included in the vaccines. Several manufacturers have been conducting clinical trials to launch norovirus vaccines on the market (43). Most of them are VLP-based vaccines, and development is also underway for an oral tablet vaccine composed of a nonreplicating adenovirus-based vector expressing the VP1 gene (44). Despite the difference in the modality, test vaccines include one GI genotype and one or more GII genotypes. Because both GI and GII consist of many genotypes, whether these vaccine candidates have efficacy against a wide variety of genotypes other than the ones included in vaccines should be carefully investigated. However, since the bivalent vaccines under development have been confirmed to induce cross-reactivity (21, 45), they are expected to be effective against a wide variety of norovirus genotypes, suggesting that cross-reactive antibodies such as CV-2F5, described in this study and others (32, 37, 41, 42), might participate in protective immunity.

## MATERIALS AND METHODS

### Norovirus strain and plasmid construction

For the production of the GI.4 Chiba VLPs, we modified pORBCVORF2,3-LAT2APV (10); the first two codons in the three consecutive Met at the initiation (Met1-Met2-Met3) for ORF2 (VP1 protein) were replaced with lysine codons (AAG) so that the VP1 protein would start at Met3. The resulting plasmid was designated pORBCVORF2,3APV-MM2KK.

For the expression of the P domain of VP1 protein from the Chiba strain (GenBank ID: AB042808), the gene fragments encoding P domains including amino acids from positions 227 to 544 were attached by overlap PCR to sequences encoding a modified poly-histidine (N11, MKDHLIHNHHKHEHAHAEH) affinity tag (46) and a small ubiquitin-related modifier (SUMO) fusion tag at the N-terminus, and then subcloned into the plasmid pCR2.1-TOPO (Thermo Fischer Scientific, Waltham, MA). The resultant plasmid expresses the N-terminal SUMO fusion protein of the P domain.

For the expression of anti-norovirus human monoclonal scFv antibodies in *Escherichia coli*, the codon-optimized CV-2F5 and CV-1A1 scFv genes were synthesized (GenScript, Piscataway, NJ) and cloned into a pCR2.1-TOPO vector with the N-terminal N11-tag and a tobacco etch virus (TEV) protease recognition site. For the CV-1A1 Fv-clasp antibody fragment expression, heavy (V_H_1-118) and light (V_L_1-111) chains of scFv CV-1A1 were individually fused with a 49-residue SARAH domain via a Gly-Ser linker as described previously (27). The SARAH domain-coding gene was kindly provided by Dr. Junichi Takagi (Osaka University, Japan). The expression plasmid of TEV protease, pRK793, was a gift from David Waugh (plasmid #8827; Addgene, Cambridge, MA) (47). For the CV-2F5 scFv, we further constructed a bacterial expression plasmid (pETSUMO-CV-2F5-Strep) in which the synthetic CV-2F5 scFv gene was flanked by the N-terminal His-tagged SUMO and the C-terminal Strep-tag.

### Preparation of VLPs

Sf9 cells (Oxford Expression Technologies, Oxford, UK) were transfected with pORBCVORF2,3APV-MM2KK together with Sapphire Baculovirus DNA (Allele Biotechnology, San Diego, CA), resulting in the production of recombinant baculoviruses, which were used for VLP production in *Trichoplusia ni* cells (Oxford Expression Technologies). VLPs were collected from culture media. The Chiba VLPs were suspended in 20 mM sodium citrate buffer (pH 4.2) and separated by isopycnic CsCl density gradient centrifugation. The VLPs were further purified by a stepwise sucrose density gradient. Purified VLPs were finally resuspended in 20 mM sodium phosphate buffer (pH 6.8) and stored at 4°C. The integrity of the VLPs was evaluated by transmission electron microscopy with uranyl acetate used as a stain.

### Preparation of P domain proteins

The Chiba P domain protein was expressed as N11-tagged SUMO fusions in an *in vitro E. coli* cell-free protein synthesis system (48). The fusion protein was immobilized by a HisTrap column (Cytiva, Piscataway, NJ), washed with 20 mM imidazole, and then eluted with 500 mM imidazole in 20 mM Tris-HCl buffer (pH 8.0) containing 0.5 M NaCl. Fusion proteins were digested by SUMO protease while dialyzing against the same buffer including 20 mM imidazole, followed by a second HisTrap chromatography. The resultant flowthrough fraction containing cleaved P domain proteins was desalted by using a HiPrep Desalting column (Cytiva) [in 20 mM Tris-HCl buffer (pH 8.0) containing 10 mM NaCl] and then subjected to anion exchange chromatography using a HiTrapQ column (Cytiva). The P domain protein was eluted by applying a NaCl gradient. The peak fraction was concentrated by using an Amicon Ultra 15 centrifugal device (MWCO 10,000) (Merck Millipore, Burlington, MA) to a protein concentration of approximately 1 mg/mL in 20 mM Tris-HCl (pH 8.0).

### Preparation of scFv and Fv-clasp antibodies

The scFv fragments fused with N-terminal N11-tag and TEV protease recognition sequences were expressed in an *E. coli* cell-free protein synthesis system as described above with the following exception: the synthesis mixture included 5 mM glutathione disulfide and 0.4 mg/mL disulfide-bond isomerase (DsbC) without reducing agent at 25°C. The synthesized proteins were purified by using the HisTrap column and then digested with TEV protease (47) and dialyzed against the same buffer including 20 mM imidazole, followed by a second HisTrap chromatography to remove cleaved His-tag and the His-tagged TEV protease. The desalted protein solution in 20 mM Tris-HCl buffer (pH 7.0) containing 80 mM NaCl was subjected to a HiTrap SP column (Cytiva) and eluted with a 0–1.0 M NaCl gradient. Eluted proteins were concentrated to 7 mg/mL.

The Fv-clasp fragments (27) derived from the CV-1A1 scFv with N-terminal N11-tag and TEV protease recognition sequences were prepared in the same manner as described above, except the synthesis mixture included 5 mM glutathione disulfide, 0.8 mg/mL DsbC, and 0.2 mg/mL Skp chaperone protein without reducing agent, and a HiTrap Q column (Cytiva) was used instead of a HiTrap SP column.

### Preparation of Strep-tagged CV-2F5 scFv antibodies

*E. coli* cells harboring pETSUMO-CV-2F5-Strep were grown on Overnight Express Instant TB Medium (Merck, Darmstadt, Germany) at 37°C overnight. Cells were harvested and lysed by shaking vigorously in the presence of 0.1 mm glass beads in phosphate-buffered saline (PBS) containing 0.1% Triton X-100, and then the supernatant was subjected to purification of Strep-tagged CV-2F5 scFv using TALON Metal Affinity resin (Takara Bio, Shiga, Japan). The N-terminal His-SUMO-tagged CV-2F5-Strep proteins bound on the resin were digested with the SUMO protease (LifeSensors, Malvern, PA), and the CV-2F5-Strep proteins without His-SUMO tag were eluted with PBS, and concentrated and desalted by using the Amicon Ultra Centrifugal Filter Unit (3K MWCO) (Merck).

### X-ray crystallography of P domain proteins

The Chiba P domain proteins were mixed with CV-2F5 scFv antibody fragments in a molar ratio of 1:1.2 and then subjected to size-exclusion chromatography. Pooled fractions including the P domain/antibody fragment complex were concentrated by ultrafiltration to approximately 8 mg/mL, then crystallized by sitting drop vapor diffusion at 25°C with a reservoir solution of 35% Tacsimate (pH 7.0) (Hampton Research, Aliso Viejo, CA). The micro-crystals that appeared were seeded in another crystallization drop and grown into larger crystals. X-ray diffraction data from the above preparations were collected by an in-house X-ray diffractometer, Rigaku FR-E (Rigaku, Tokyo). The diffraction data were processed with the HKL-2000 program (49), and the structure was solved by molecular replacement using the Phaser program (50) in the Phenix suite (51), with the P domain of the GI.4 (PDB ID: 8J5B) and scFv (PDB ID: 5C6W) as the search models. The refinement was conducted using the Phenix programs, and the structure was manually rebuilt with the Coot program (52). Data collection and refinement statistics are presented in Table 2. The Waals (Altif Laboratories, Tokyo) and the UCSF ChimeraX (53, 54) were also used for structure visualization.

**TABLE 2:** X-ray data collection and refinement statistics for the complex of the Chiba P domain and the CV-2F5 scFv

Data collection	
Spacegroup	*C2*
Resolution range (Å)	41.35–2.70 (2.80–2.70)^*a*^
Unit cell	
*a*, *b*, *c* (Å)	176.67, 102.25, 90.79
α, β, γ (°)	90.00, 109.93, 90.00
Total reflections	156,139
Unique reflections	41,894
Completeness (%)	99.9 (99.3)
Redundancy	3.7 (3.4)
*I*/σ(*I*)	14.0 (2.1)
*R*_meas_	0.111 (0.664)

Refinement statistics	
Resolution (Å)	2.70
*R*_work_	0.184
*R*_free_	0.259
R.m.s. deviations	
Bond lengths (Å)	0.008
Bond angles (°)	1.05
Average *B*-factor (Å^2^)	41.7
Ramachandran plot (%)	
Favored	94.49
Allowed	5.36
Outliers	0.15
PDB ID	8I5L

^*a*^ Numbers in parentheses refer to the highest resolution shell.

### Cryo-EM data acquisition and image processing

The purified VLPs were mixed with the Fv-clasp fragments in a 1:5 molar ratio and incubated for 60 min on ice to form the VLP/Fv-clasp complexes. Four microliters of the complex was applied onto a glow-discharged Quantifoil holey carbon grid (R1.2/1.3, 300 mesh) (Quantifoil Micro Tools, Jena, Germany). The grid was rapidly plunged into liquid ethane using a Vitrobot Mark IV (Thermo Fisher Scientific), with blotting for 3 s at 4°C with 100% humidity.

The flow of the cryo-EM analysis is summarized in Fig. S6. Data were acquired with the use of a Tecnai Arctica transmission electron microscope (Thermo Fisher Scientific) operated at 200 kV with a K2 Summit direct electron detector (Gatan, Pleasanton, CA) at a nominal magnification of 23,500×, which resulted in 1.47 Å/pixel at the specimen level. All of the data were collected as movies with 40 subframes with a total electron dose of 50 e^-^/Å^2^. The 1,809 movies were imported into the cryoSPARC package (v.3.3.2) (55), followed by motion correction and contrast transfer function (CTF) estimation. Then, 47,186 particles selected by 2D classification were input to execute *ab initio* 3D reconstruction with icosahedral symmetry, followed by further homogeneous refinement and CTF refinement, resulting in the overall structure at a resolution of 3.0 Å.

Next, by running symmetry expansion, 2,827,500 particles were generated and subjected to local refinement and 3D classification between asymmetric units of the particles. As a result, particles with two Fv-clasp fragments attached to the A/B dimer of the VP1 proteins were selected, and the final map was obtained by local refinement.

We used the structures of GI.1 Norwalk strain VLP (PDB ID: 6OUT) and P20.1 Fv-clasp (PDB ID: 5XCQ) as the starting models for model building and refinement against the electron density map. The cryo-EM model was docked into the electron microscopy density map using ChimeraX (53, 54), followed by iterative manual adjustment and rebuilding in Coot (52) and phenix.real_space_refine in Phenix (51, 56). For cross-validations, the final model was refined against one of the half-maps generated by 3D auto-refine, and the model vs. map Fourier shell correlation curves were generated in the Comprehensive Validation module in Phenix. The final refinement statistics are provided in Table S1.

### Sandwich ELISA using human monoclonal antibody fragments

Since the CV-2F5 scFv has a Strep-tag at its C-terminus, 50 µL of 20 µg/mL (0.7 µM) solution in PBS was added to each well of a Strep-Tactin-coated 8-well strip (IBA Lifesciences GmBH, Göttingen, Germany), followed by 1-h incubation at 37°C. As for the CV-1A1 Fv-clasp and scFv, each well of a 96-well ELISA microplate (MICROLON 600, high binding; Greiner Bio-One International GmbH, Frickenhausen, Germany) was coated using 50 µL of 0.7 µM solution prepared in 50 mM carbonate buffer (pH 9.6). After washing the wells, 50 µL of 400 µM Chiba or Vancouver VLPs was added and twofold serial dilutions were made. Wells to which VLPs were not added were used as controls (0 nM VLPs). After incubation at 37°C for 2 h, to detect each VP1 protein bound to antibody, rabbit antisera raised against Chiba VLPs (laboratory stock) or Vancouver VLPs (Scrum, Tokyo) diluted in dilution buffer (Dulbecco’s PBS (D-PBS) containing 0.5% Tween 20 and 5% skim milk) at 1:3,000 were added, followed by incubation at 37°C for 1 h. Then, horseradish peroxidase (HRP)-conjugated donkey anti-rabbit IgG (Abcam, Cambridge, MA) was added and the microplate was incubated at 37°C for 1 h. ABTS (Roche, Basel, Switzerland) was used as the HRP substrate. The absorbance at 405 nm was measured; the absorbance at 630 nm was simultaneously recorded as a reference. ∆OD indicates the values for the absorbance at 630 nm subtracted from those for the absorbance at 405 nm. The KaleidaGraph software package (Hulinks, Tokyo) was used for data analysis.

### ELISA-based assay for the characterization of antibody fragments

The BSA conjugates of LNFDH I (lacto-N-difucohexaose I, Lewis b hexasaccharide) (Dextra Laboratories, Reading, UK) were solved in phosphate buffer (0.125 M KH_2_PO_4_, 0.125 M Na_2_HPO_4_, pH 6.8) at 1 mg/mL and stored at −80°C. The conjugates were diluted in 50 mM sodium carbonate buffer (pH 9.6) at 20 µg/mL, and a 96-well ELISA microplate (Greiner Bio-One) was coated with 50 µL/well of the diluents and left at 4°C overnight. After washing with D-PBS containing 0.05% Tween 20, the microplate was blocked by dilution buffer (see above) for 1 h at 37°C. After washing, 25 µL of the dilution buffer was poured into each well, and 25 µL of each of the additives [8 µM CV-1A1 Fv-clasp, CV-1A1 scFv, and CV-2F5 scFv; 0.5 µM anti-Lewis b monoclonal antibody 2–25LE and anti-Lewis a monoclonal antibody 7LE (Novus Biologicals, Centennial, CO); and 16 mM free Lewis b tetrasaccharide (Elicityl, Crolles, France)] in dilution buffer was added to the wells in column 2 of the microplate, and twofold serial dilutions of the additives were made to column 10. The wells in column 11 did not contain any additives. Then, 25 µL of 0.2 µM Chiba or Vancouver VLPs in dilution buffer was added, followed by incubation at 37°C for 1 h. The subsequent processes were done in the same manner as described in the previous section.

### Effects of antibody fragments on morphology of VLPs

The Chiba and Vancouver VLPs were mixed with antibody fragments in a molar ratio of 1:5 (VP1:antibody), and incubated at 4°C for 3 h. Then VLPs were observed under a transmission electron microscope.

## Data Availability

The structural data obtained in this study were deposited in the Protein Data Bank (PDB) and Electron Microscopy Data Bank (EMDB) with the accession numbers PDB ID: 8I5L for the GI.4 P domain/CV-2F5 scFv fragment complex, and EMD-36223 and PDB ID: 8JG5 for the GI.4 VLP/CV-1A1 Fv-clasp complex. All other data from the current study are available from the corresponding authors upon reasonable request.

## SUPPLEMENTAL MATERIAL

Supplemental figures jvi.00197-24-s0001.pdf Figures S1 to S6.

Table S1 jvi.00197-24-s0002.docx Cryo-EM data.

ASM does not own the copyrights to Supplemental Material that may be linked to, or accessed through, an article. The authors have granted ASM a non-exclusive, world-wide license to publish the Supplemental Material files. Please contact the corresponding author directly for reuse.
