# Supplementary material for: Structural analyses of the GI.4 norovirus by cryo-electron microscopy and X-ray crystallography revealing binding sites for human monoclonal antibodies
Source: J Virol. 2024 Apr 9;98(5):e00197-24. doi: 10.1128/jvi.00197-24 (PMC11092324; doi:10.1128/jvi.00197-24)
Supplement: Supplemental figures — Figures S1 to S6. [file jvi.00197-24-s0001.pdf]

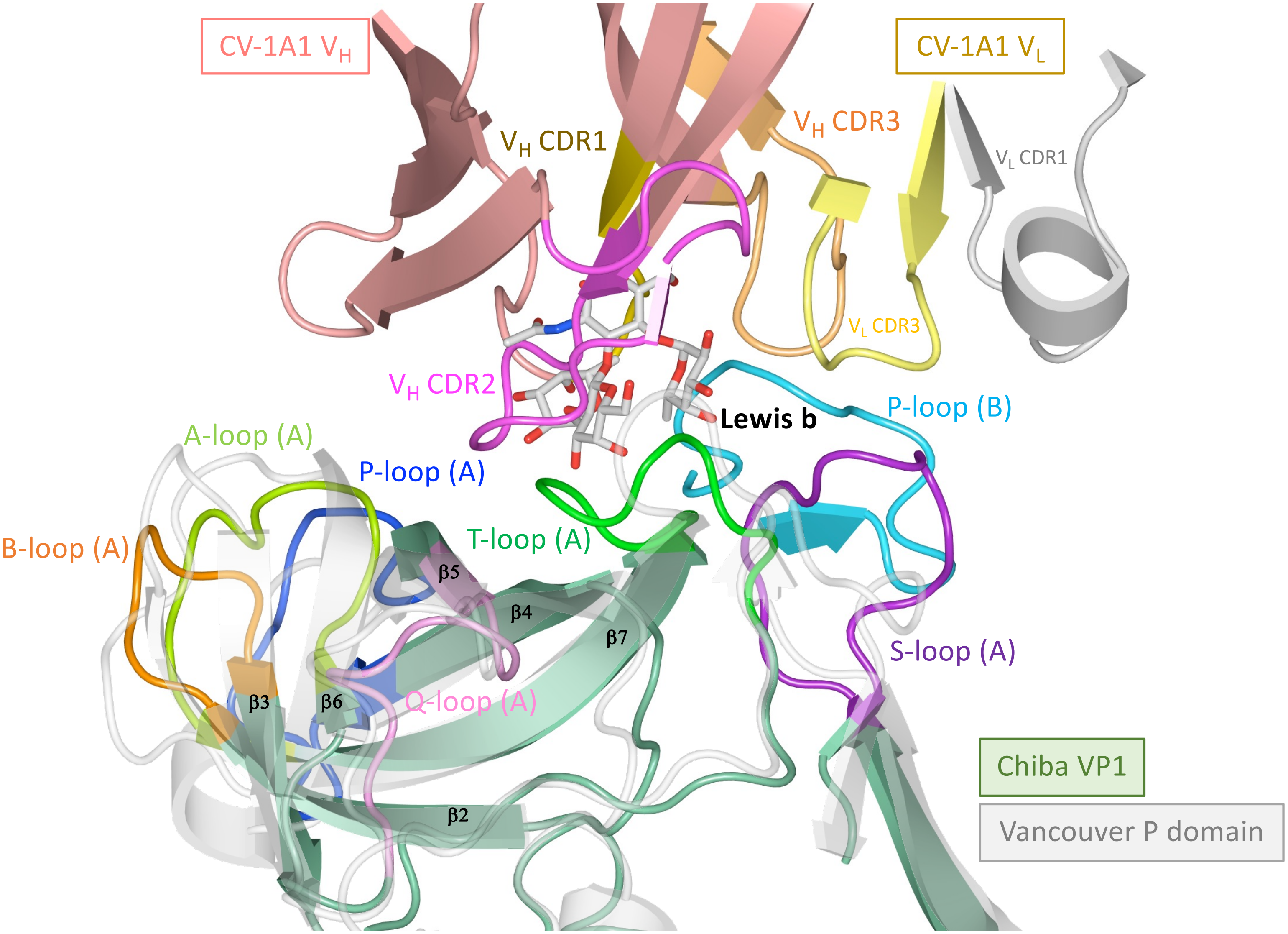

Supplementary Figure S1. The superposition of the CV-1A1 Fv-clasp-bound GI.4 Chiba VP1 on the Lewis b-bound GI.9 Vancouver P-domain protein. The structure of the CV-1A1 Fv-clasp-bound Chiba VP1 was overlaid on the structure of the Lewis b-bound Vancouver P-domain protein [28]. The Chiba VP1 and the Vancouver P-domain are colored *green* and *pale gray*, respectively. In the Chiba VP1, loop regions are indicated with their respective names. The V_H CDR2 of the CV-1A1 Fv-clasp overlaps with the binding site for the Lewis b antigen.

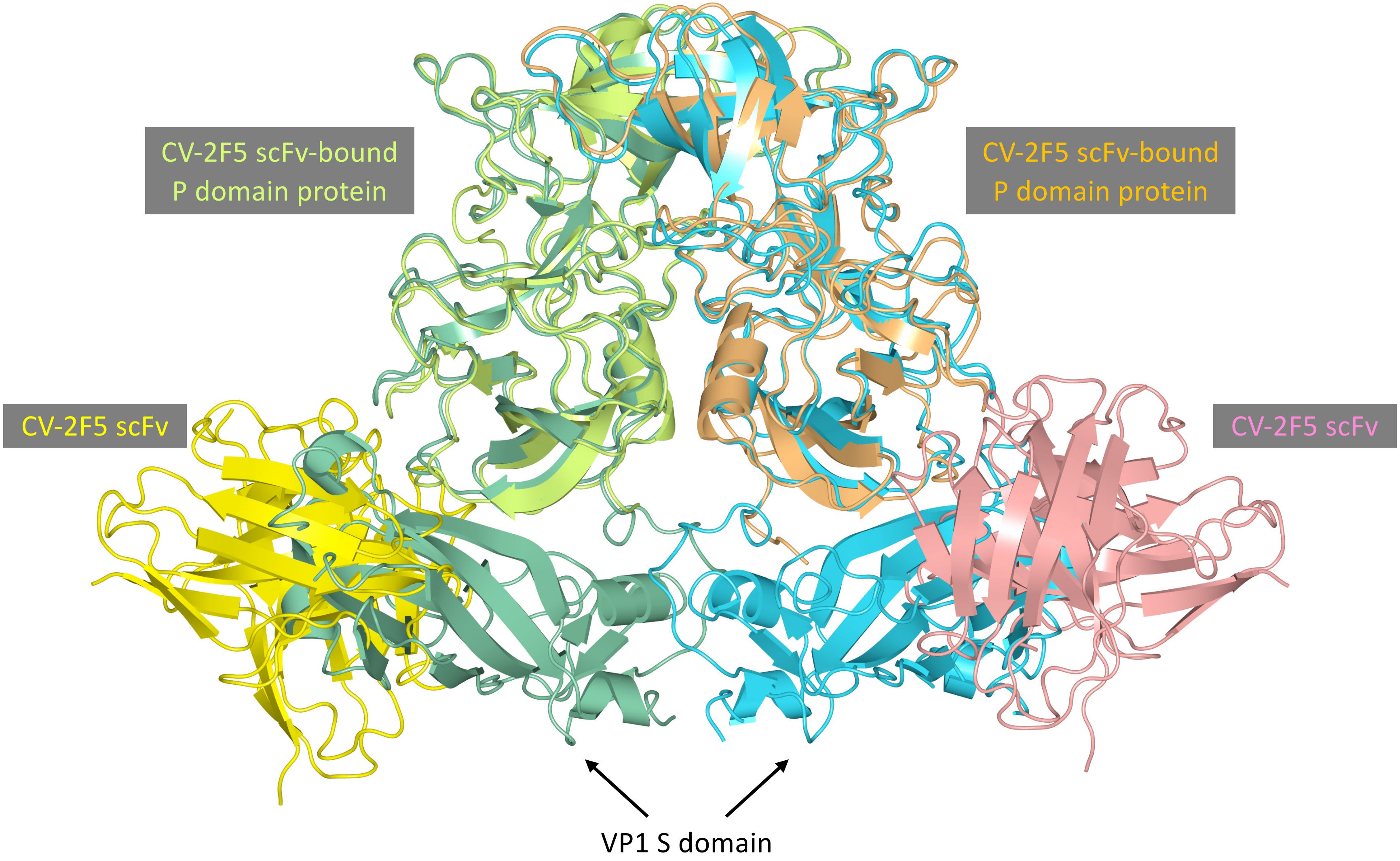

Supplementary Figure S2. The superposition of the CV-2F5 scFv-bound P domain protein on the VP1 protein. The structure of the CV-2F5 scFv-bound Chiba P domain protein (*yellow green* and *orange*) was overlaid on the VP1 protein (*green* and *cyan*) determined by cryo-EM (see Fig. 3). The CV-1A1 Fv-clasp is not shown in this figure. The CV-2F5 scFv (*yellow* and *pale red*) clashes with the S domain of the VP1 protein.

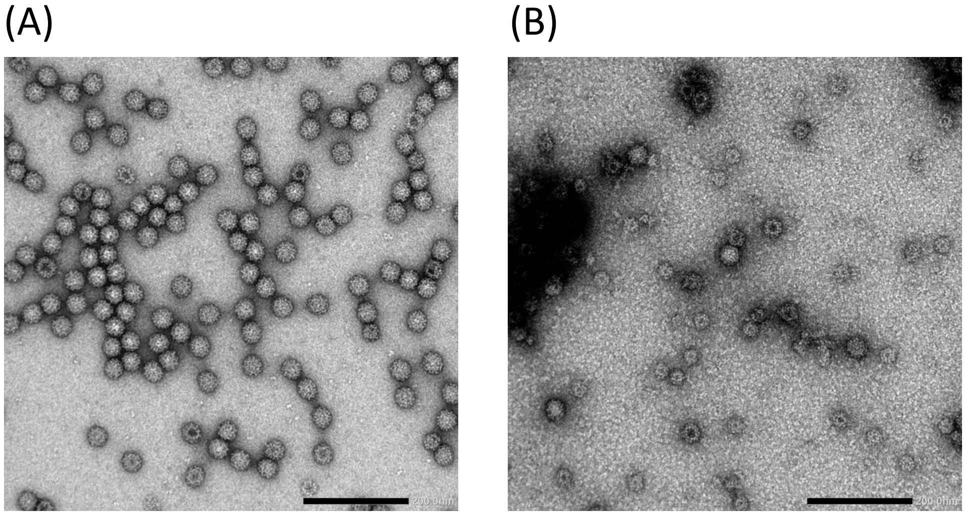

Supplementary Figure S3. Effects of the CV-2F5 scFv antibody fragments on morphology of Vancouver VLPs were visualized by transmission electron microscopy. (A) VLPs without CV-2F5, (B) VLPs mixed with CV-2F5.

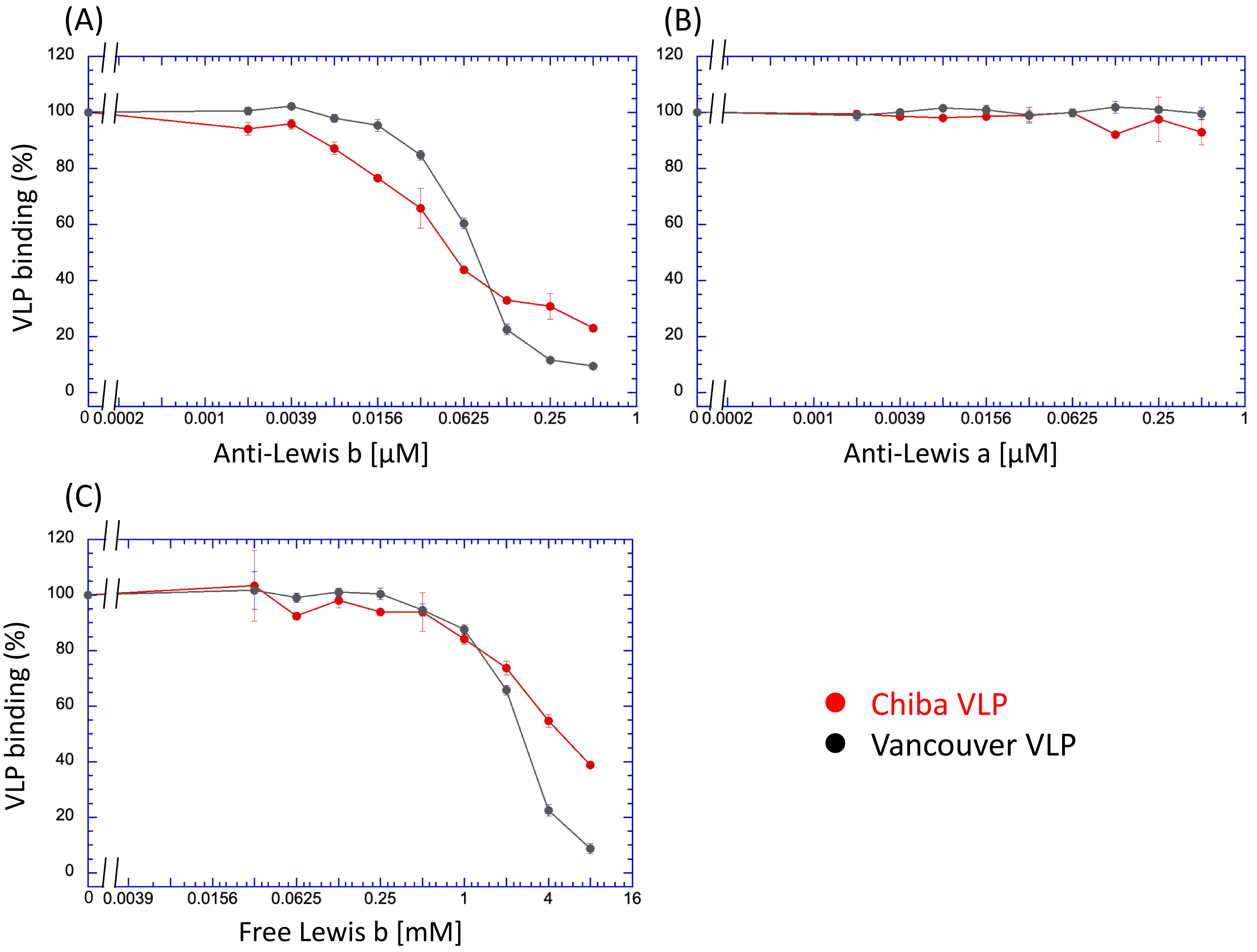

Supplementary Figure S4. Effects of anti-Lewis saccharide antibodies and free Lewis b tetrasaccharides on VLP binding to Lewis b saccharide. Microplates were coated with the BSA conjugates of LNFDH I, which contains Lewis b tetrasaccharide, as described under the Materials and Methods, and VLPs (final concentration of 0.1 μM) and the indicated concentrations of antibodies (anti-Lewis b monoclonal antibody (A), or anti-Lewis a monoclonal antibody (B)), or free Lewis b tetrasaccharide (C) were added, followed by detection of bound VP1 proteins using the respective rabbit antisera.

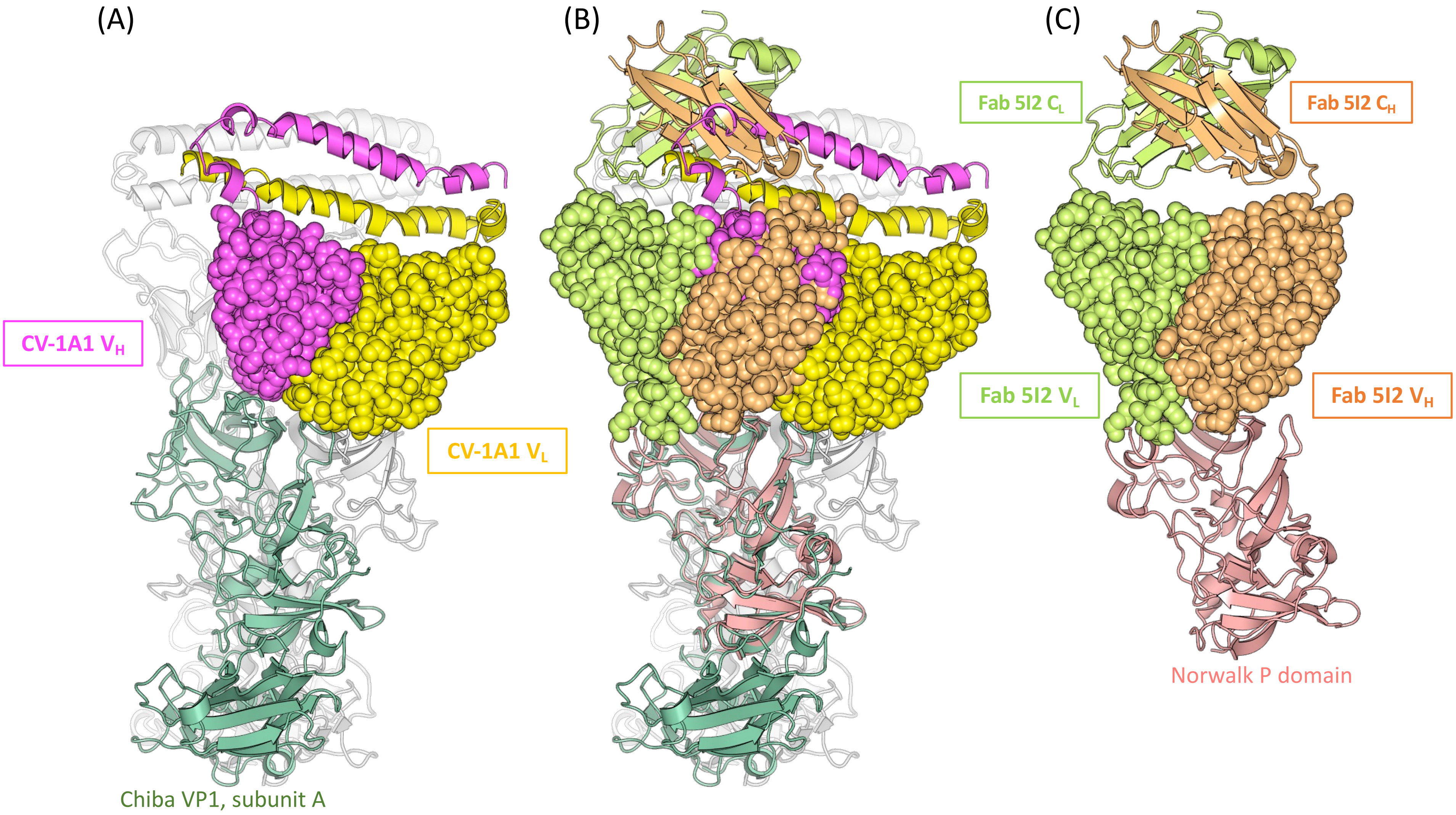

Supplementary Figure S5. The differences in binding mode between the CV-1A1 and the IgA Fab 5I2. Panel (A) shows the sideview of the CV-1A1 Fv-clasp-bound Chiba VP1 dimer (cf. the front view shown in Fig. 1B). The A subunit of the VP1 dimer is colored *green*. The V_H and V_L domains of the CV-1A1-Fv-clasp mainly bound to the A subunit are colored *magenta* and *yellow*, respectively, and are displayed in a space-filling model. Both the B subunit of the VP1 dimer and the Fv-clasp mainly bound to the B subunit are colored *gray* for clarity. On panel (B), the structure of the CV-1A1 Fv-clasp-bound Chiba VP1 was overlaid on the structure of the IgA Fab 5I2-bound Norwalk P-domain protein [30]. The sideview of the Fab 5I2-bound Norwalk P domain monomer is shown in panel (C). The Norwalk P domains are colored *pale red*, and the heavy chain and light chain of the Fab 5I2 are colored *orange* and *yellow-green*, respectively. The V_H and V_L domains of the Fab 5I2 are also displayed in a space-filling model.

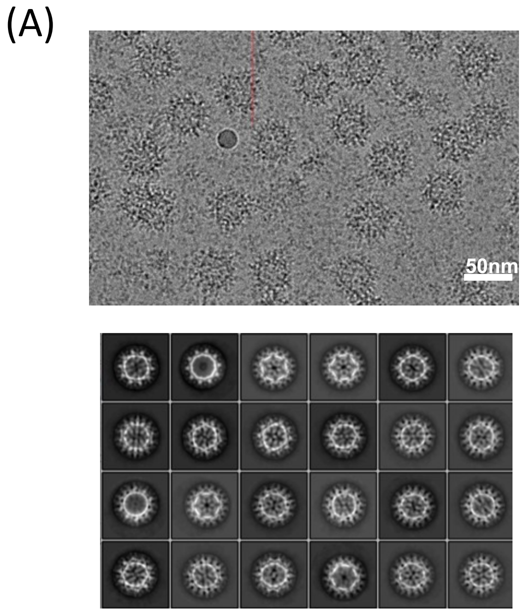

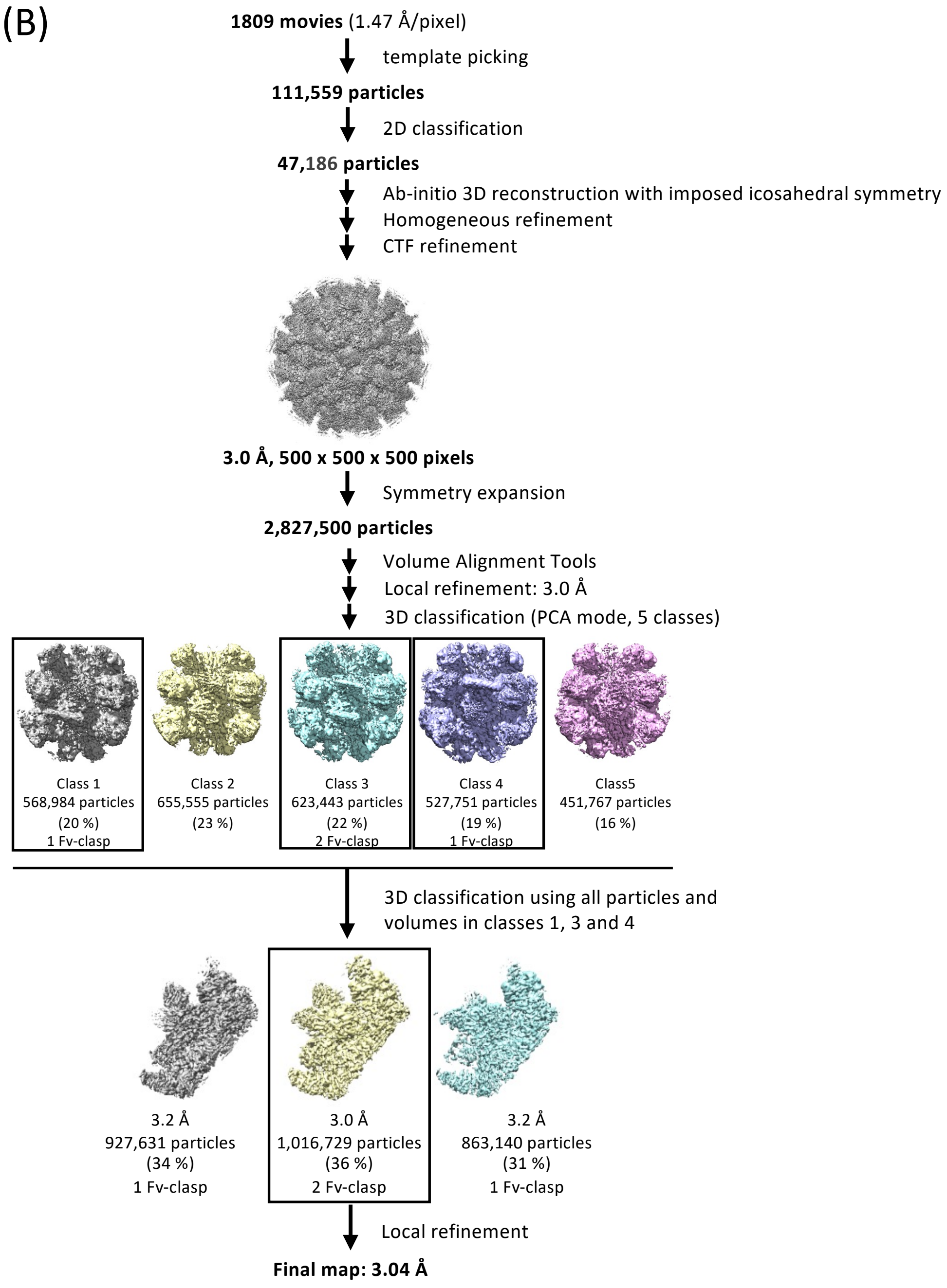

Supplementary Figure S6. The flow chart of the cryo-EM data processing for the Chiba VLP/CV-1A1 Fv-clasp complexes. **(A)** Electron microgram of the CV-1A1 Fv-clasp-bound VLPs. *Upper panel:* a TEM image of the complexes. *Lower panel:* examples of selected particles. **(B)** The cryo-EM data of the complexes were processed with the cryoSPARC package [55] as described in the Materials and Methods section.
