# Supplementary material for: Structural analyses of the GI.4 norovirus by cryo-electron microscopy and X-ray crystallography revealing binding sites for human monoclonal antibodies
Source: J Virol. 2024 Apr 9;98(5):e00197-24. doi: 10.1128/jvi.00197-24 (PMC11092324; doi:10.1128/jvi.00197-24)
Supplement: Table S1 — Cryo-EM data. [file jvi.00197-24-s0002.docx]

**Supplementary Table S1.** Cryo-EM data collection, refinement, and validation statistics.

| **PDB ID** | **8JG5** |
| --- | --- |
| **EMDB ID** | **EMD-36223** |
| ***Data collection and processing*** |  |
| Magnification | 23,500 |
| Voltage (kV) | 200 |
| Electron exposure (e^–^/Å^2^) | 50 |
| Defocus range (µm) | −0.8 to −1.6 |
| Pixel size (Å) | 1.47 |
| Symmetry imposed | Icosahedral |
| No. of initial particle images | 111,559 |
| No. of final particle images | 47,125 |
| Map resolution (Å) | 3.04 |
| FSC threshold | 0.143 |
| ***Refinement*** |  |
| Model resolution (Å) | 3.04 |
| FSC threshold | 0.5 |
| Model composition |  |
| No. of non-hydrogen atoms | 16,057 |
| No. of protein residues | 2,086 |
| B factors (Å^2^) |  |
| Protein | 160.79 |
| R.m.s. deviations |  |
| Bond lengths (Å) | 0.004 |
| Bond angles (°) | 0.655 |
| Validation |  |
| MolProbity score | 1.91 |
| Clash score | 15.07 |
| Poor rotamers (%) | 0.39 |
| Ramachandran plot |  |
| Favored (%) | 96.55 |
| Allowed (%) | 3.45 |
| Disallowed (%) | 0 |
